# Evaluation of point-of-care haemoglobin measurement accuracy in surgery (PREMISE) and implications for transfusion practice: a prospective cohort study

**DOI:** 10.1016/j.bja.2024.09.033

**Published:** 2025-01-09

**Authors:** Karine Brousseau, Leah Monette, Daniel I. McIsaac, Christopher Wherrett, Ranjeeta Mallick, Aklile Workneh, Tim Ramsay, Alan Tinmouth, Julie Shaw, Justin Presseau, Julie Hallet, François M. Carrier, Dean A. Fergusson, Guillaume Martel

**Affiliations:** 1 Department of Surgery, The Ottawa Hospital, University of Ottawa, Ottawa, ON, Canada; 2 Clinical Epidemiology Program, Ottawa Hospital Research Institute, Ottawa, ON, Canada; 3 Department of Anesthesiology and Pain Medicine, The Ottawa Hospital, University of Ottawa, Ottawa, ON, Canada; 4 Department of Medicine, The Ottawa Hospital, University of Ottawa, Ottawa, ON, Canada; 5 Eastern Ontario Regional Laboratories Association, Ottawa, ON, Canada; 6 Department of Pathology and Laboratory Medicine, University of Ottawa, Ottawa, ON, Canada; 7 Département d'anesthésie, Centre Hospitalier de l'Université de Montréal, Université de Montréal, Montreal, QC, Canada; 8 Division de soins critiques, Département de médecine, Centre Hospitalier de l'Université de Montréal, Université de Montréal, Montreal, QC, Canada; 9 Sunnybrook Research Institute, Sunnybrook Health Sciences Centre, Toronto, ON, Canada

**Keywords:** haemoglobin, method comparison, noncardiac surgery, point-of-care, transfusion

## Abstract

**Background:**

Point-of-care testing devices to measure haemoglobin (Hgb) frequently inform transfusion decision-making in surgery. This study aimed to examine their accuracy in surgery, focusing on Hgb concentrations of 60–100 g L^−1^, a range with higher potential for transfusion.

**Methods:**

This was a prospective diagnostic cohort study focused on method comparison, conducted at two academic hospitals. Consecutive patients undergoing noncardiac surgery and requiring point-of-care Hgb measurements were eligible. Hgb concentrations from arterial and central venous blood samples were measured concurrently using three devices and compared with laboratory Hgb. The primary outcome was individual pairwise comparisons between point-of-care and laboratory Hgb values; agreement was determined based on a threshold of within 4 g L^−1^. The primary analysis consisted of computing limits of agreement.

**Results:**

A total of 1735 intraoperative blood samples were collected (1139 participants); 680 samples had a laboratory Hgb <100 g L^−1^. The limits of agreement among those with Hgb <100 g L^−1^ were –9.5 to 8.0 g L^−1^ for HemoCue®, –16.2 to 11.5 g L^−1^ for i-STAT®, and –14.7 to 40.5 g L^−1^ for Rad-67®. HemoCue was associated with a 5.8% incidence of potentially clinically significant transfusion error, whereas i-STAT and Rad-67 were associated with 25.3% and 28.2%, respectively. HemoCue yielded Hgb measurements within 10 g L^−1^ in 98% of intraoperative blood samples.

**Conclusions:**

No point-of-care Hgb device demonstrated limits of agreement that were smaller than the agreement difference of 4 g L^−1^. Despite this, HemoCue can be safely used to inform transfusion decisions in surgery, given its error probability of <4% in transfusion scenarios.

**Editor's key points**

• Point-of-care measurements of haemoglobin concentration frequently inform transfusion decision-making in surgery.
• The authors conducted a prospective diagnostic cohort study comparing three point-of-care haemoglobin measurement devices with laboratory measurements at two academic hospitals in patients undergoing noncardiac surgery.
• HemoCue® was associated with a 5.8% incidence of potentially clinically significant transfusion errors, whereas i-STAT and Rad-67 were associated with 25.3% and 28.2%, respectively.
• Point-of-care Hgb measurements can vary widely and lead to considerable differences when compared with laboratory Hgb results, and none of the devices studied can be considered interchangeable with laboratory measurements.

Red blood cell (RBC) transfusions are common during surgery and can be life-saving,^1^ but when administered without an appropriate clinical indication, they provide no benefit to patients and can potentially cause harm.^2^ Transfusion decision-making in the intraoperative setting is a complex and dynamic process,^3^ which is further hampered by limited available evidence and practice guidelines for surgical patients.^4^ Unsurprisingly, there is widespread evidence of variability in intraoperative transfusion practice.^5^^,^^6^

Previous studies have reported an important relationship between haemoglobin (Hgb) or haematocrit (Hct) measurement and intraoperative transfusion decision-making.^7^^,^^8^ Practise guidelines recommend measuring Hgb or Hct as part of an approach to monitor for intraoperative anaemia^4^ and suggest that RBC transfusions are rarely indicated with Hgb concentrations >100 g L^−1^.^9^ Although intraoperative Hgb concentrations were previously measured using a blood sample sent to a central laboratory (lab-Hgb), point-of-care testing devices measuring Hgb (POCT-Hgb) are now commonly used, owing to ease of use and rapid results delivery in a context where Hgb concentrations can change rapidly and drastically.^10^ Despite their widespread use, there is limited evidence on the accuracy of POCT-Hgb devices in the intraoperative setting. Most studies examining the accuracy of POCT-Hgb devices were conducted outside of the operating room or in small surgical studies within specific subgroups, providing conflicting conclusions.^11^^,^^12^ Data are particularly limited at Hgb concentrations of 60–100 g L^−1^, where RBC transfusions might be clinically indicated but remain nuanced.^7^^,^^11^^,^^13^

The primary objective of the PREMISE (point-of-care haemoglobin accuracy and transfusion outcomes in noncardiac surgery) study was to evaluate the real-world accuracy of three commonly used classes of intraoperative POCT-Hgb technology in noncardiac surgery with a specific focus on Hgb measurements <100 g L^−1^.

## Methods

The PREMISE study was a prospective diagnostic cohort study comparing three POCT-Hgb devices with central laboratory testing. It was conducted at two tertiary-care academic hospitals in Ottawa, ON, Canada, between March 2021 and July 2022. The study protocol has been published.^14^ This study was approved by the Ottawa Health Science Network—Research Ethics Board (OHSN-REB 20200731-01H). A waived consent model was approved given the observational nature of this study.

Study results are reported following STARD 2015 guidelines (Supplementary Table S1)^15^ and reporting standards for agreement analyses.^16^

### Participants

The study population consisted of consecutive adult patients undergoing major noncardiac surgery during recruitment hours (Monday–Friday, 10:00 to 19:00) and requiring intraoperative blood analysis using a POCT-Hgb device. Patients were excluded if they were undergoing cardiac or obstetrical surgery, if they did not have general or neuraxial anaesthesia, or if they did not have an arterial or central venous catheter. Repeated measurements from patients during the same operation were allowed.

### Test methods

All intraoperative POCT-Hgb blood draws by the anaesthesiology team were included in the study and concurrently analysed using three POCT-Hgb devices and a central laboratory test. The POCT-Hgb devices used were HemoCue® (HemoCue AB, Ängelholm, Sweden), which uses a spectrophotometric method to measure Hgb^17^; i-STAT™ (Abbott Laboratories, Abbott Park, IL, USA), which uses a conductometric method to measure Hct and then converts it to Hgb using a proportionality constant^18^; and Rad-67™ pulse CO-Oxymeter® (Masimo, Irvine, CA, USA), which uses a noninvasive multiwavelength finger sensor.^19^ A POCT-Hgb device (i-STAT or HemoCue) was chosen and used by the requesting anaesthesiologist for clinical purposes and as part of standard care, whereas all other POCT-Hgb and lab-Hgb results were not shared with the clinical team and were used for research purposes only.

### Point-of-care testing devices measurement of haemoglobin (index tests)

Approximately 3 ml of whole blood was collected from the arterial or central venous catheter for use with HemoCue and i-STAT. Hgb was simultaneously measured with Rad-67 using a finger sensor. POCT-Hgb measurements were performed within 5 min of blood collection. The manufacturers' instructions on handling, training, storage, calibration, quality control, and institutional POCT quality assurance policies were followed for each device.

### Laboratory measurement of haemoglobin (reference standard)

The remaining whole blood sample was collected using an ethylenediaminetetraacetic acid (EDTA) vacuum collection tube and processed by trained staff from the central laboratory using a Sysmex XE-2100 analyser (Sysmex Corporation, Kobe, Japan), which uses the standard haemoglobincyanide method, the widely accepted standard for Hgb measurement. Laboratory staff did not have access to the POCT-Hgb results.

## Statistical analysis

Statistical analysis was performed using SAS 9.4 (SAS Institute, Cary, NC, USA) based on a statistical analysis plan published with the study protocol.^14^ The primary analysis consisted of examining continuous agreement of individual pairwise comparisons between each POCT-Hgb device and the lab-Hgb, first for the group with lab-Hgb 60–100 g L^−1^ and then for the whole population. This was carried out using the limit of agreement (LOA) method adjusted for repeated measurements, where the true value is assumed to vary, with 95% confidence intervals (CIs).^20^, ^21^, ^22^ Visual examination of the pairwise comparisons was performed using plots to ensure that assumptions about the distribution of the data were met for the LOA analyses, including normal distribution of the differences, similarity of agreement over the range of measurements and over time, and absence of relationships between the within-subject variability and Hgb measurements.^20^

Bland–Altman plots of the difference between POCT-Hgb and lab-Hgb measurements against the lab-Hgb measurement were generated.^20^^,^^21^ Although it is recommended to use the average of two measurements for the *x*-axis to account for possible variability in the reference test when the true value is unknown,^21^^,^^23^ the lab-Hgb was used on the *x*-axis, as it is widely accepted as the clinical gold standard.^24^ Folded empirical distribution function curves were plotted as a nonparametric method for complementary visualisation of the distribution of differences.^13^^,^^25^

The definition provided by the Institute for Quality Management in Healthcare (IQMH), the proficiency testing provider for all licensed laboratories in the Canadian province of Ontario, was used. This defines the allowable difference of within 4 g L^−1^ from lab-Hgb values <100 g L^−1^ and within 5% for lab-Hgb values ≥100 g L^−1^.^26^ Additional agreement thresholds for an allowable difference of within 10 g L^−1^ and within 7% from the lab-Hgb value were examined as secondary analyses, as these have been reported in the literature^27^ or recommended in guidelines by the Clinical Laboratory Improvement Amendments.^28^^,^^29^

Subgroup analyses were conducted to examine the effect of sex on LOA. Sensitivity analyses were conducted to examine the influence of outliers (determined by visual inspection of the Bland–Altman plots), repeated measurements, and protocol deviations on LOA by removing the relevant blood samples from analysis (Supplementary Table S2). Although it is advisable to keep outlier data within LOA analyses, their influence on LOA should be examined.^21^

Error grid analysis was conducted as a secondary analysis.^27^^,^^30^ The results of each POCT-Hgb device were plotted against the lab-Hgb measurement on a grid consisting of three different zones representing low (A), moderate (B1/B2), and high (C) risk for potentially clinically relevant transfusion errors.^27^ Zones B1 and B2 represent the potential for under-transfusion (POCT-Hgb > lab-Hgb) and over-transfusion (POCT-Hgb < lab-Hgb), respectively. A modification to the grid suggested by Morey and colleagues^27^ was produced to adjust the narrow band around the unity line within lab-Hgb values of 60–100 g L^−1^ to allow a difference of within 4 g L^–1^^26^ instead of within 10%.^27^ The proportions of pairs of measurements falling within each zone were computed.

Disagreement proportions in individual pairwise comparisons were generated as dichotomous outcomes, using the three different agreement thresholds described above with 95% CI adjusted for repeated measurements using the Wilson interval method with continuity correction.^31^ Cohen's kappa coefficients with 95% CI were computed to further analyse the pairwise comparisons as a dichotomous outcome. Hgb transfusion triggers were chosen to examine the probabilities of agreement between POCT-Hgb and lab-Hgb based on the potential to transfuse at various Hgb thresholds beyond chance alone.^27^^,^^32^^,^^33^ The strength of agreement was determined using the ranges of agreement defined by Landis and Koch.^32^^,^^33^

Indeterminate POCT-Hgb or lab-Hgb measurements were considered missing data. Missing lab-Hgb measurements were completely excluded from the analysis. Indeterminate POCT-Hgb measurements were removed from the analysis for that specific POCT-Hgb device.

### Sample size

As this is an observational study, a convenience sample size of 1750 individual Hgb measurements was planned, yielding 652 Hgb measurements with a lab-Hgb <100 g L^−1^ based on internal unpublished data showing that ∼38% of POCT-Hgb blood samples in the operative setting have lab-Hgb <100 g L^−1^. The sample size of 652 Hgb measurements was determined to be needed to fit a stable predictive logistic regression model with up to 15 independent predictors, assuming a c-statistic of 0.75, a prevalence of 0.5 (i.e. ∼50% of POCT-Hgb obtained with i-STAT would fall outside of the acceptable difference of within 4 g L^−1 26^), based on methods described by Riley and colleagues.^34^ This sample size was formally powered for the secondary outcome of predictive modelling, but was also calculated to have >90% power for the primary outcome of agreement for each device, assuming mean differences and standard deviations from Ahn and colleagues^11^ and using the method proposed by Shieh.^35^ Secondary objectives will be reported separately.

## Results

A total of 1135 surgical patients were included in the study, and 1735 individual Hgb measurements were collected, of which 680 had a lab-Hgb <100 g L^−1^ (Supplementary Fig. S1). A total of 732 patients had a single measurement, whereas 403 had multiple measurements during surgery, ranging from 2 to 9 measurements each. Only 1312 (75.6%) measurements from Rad-67 were included in the analysis as other measurements were missing for this device, either because no finger was available during surgery as the hands were tucked under drapes or because the device failed to register a Hgb measurement because of low peripheral perfusion. Baseline participant characteristics and intraoperative characteristics are shown in Table 1, with descriptive statistics computed at the individual participant level (*n*=1135). No adverse event resulted from performing the index tests or reference standard.

**Table 1:** Baseline patient characteristics. CCI, Charlson Comorbidity Index; RBC, red blood cell. ∗Median and range presented. ^†^Mean and sd presented for continuous measurements. ^‡^Median and interquartile range presented for continuous measurements. ^¶^Other types of surgery include urology (9.4%); thoracic surgery (6.2%); ears, nose, and throat surgery (3.0%); plastic surgery (2.0%); and non-obstetrical gynaecology (1.8%). ^§^Of those having received tranexamic acid (TXA), 69 (11.3%) participants were co-enrolled in a blinded RCT with a 1:1 allocation of TXA *vs* placebo. Of those, ∼50% should have received TXA. The study allocation was still blinded at the time of analysis. ^||^The numbers of each vasopressor do not add to the group total as some patients received more than one type of vasopressor during surgery. ^#^Other vasopressors used included vasopressin (4.6%), epinephrine (2.6%), and milrinone (1.2%).

	Values, *n* (%) or mean (sd) (*n*=1135)
Baseline patient characteristics	
Age (yr)	66 (14–98)∗
Female sex	489 (43.1)
Body mass index (kg m^−2^) (*n*=1108)	28.3 (7.2)^†^
Preoperative haemoglobin (g L^−1^) (*n*=1111)	121 (23.5)^†^
CCI	2 (1–4)^‡^
Localised solid tumour	467 (41.2)
Peripheral vascular disease	238 (21.0)
Myocardial infarction	231 (20.4)
Uncomplicated diabetes mellitus	228 (20.1)
CCI score of 0	155 (13.7)
Preoperative chemotherapy	156 (13.8)
Preoperative radiation treatment	95 (8.4)
Type of surgery	
General surgery	229 (20.2)
Vascular surgery	228 (20.1)
Orthopaedic surgery	226 (19.9)
Neurosurgery	198 (17.4)
Other^¶^	254 (22.4)
Priority of surgery	
Elective	678 (59.7)
Urgent	457 (40.3)
Intraoperative characteristics	
Anaesthesia type	
General anaesthesia	891 (78.5)
Neuraxial anaesthesia	27 (2.4)
General and neuraxial anaesthesia	212 (18.7)
Intravenous sedation only	5 (0.44)
Estimated blood loss (ml) (*n*=1086)	550 (300–1000)^‡^
Crystalloid infusion (ml) (*n*=1091)	2688 (1900–3545)^‡^
Intraoperative RBC transfusion	346 (30.5)
Anticoagulation administration	
Intravenous heparin	249 (21.9)
Prophylactic (subcutaneous) heparin	199 (17.6)
Blood-sparing strategies used	
Tranexamic acid	612 (53.9)^§^
Protamine	203 (17.9)
Cell salvage	75 (6.6)
Vasopressor use	1069 (94.2)^||^
Phenylephrine	913 (80.4)
Ephedrine	565 (49.8)
Norepinephrine	324 (28.6)
Other^#^	96 (8.5)

### Test results

The LOAs and 95% CIs obtained for each POCT-Hgb device are presented in Table 2 and the Bland–Altman plots in Supplementary Figure S2, for the population at a higher risk of blood transfusion (lab-Hgb <100 g L^−1^) and for the full population. Mean differences were negative for HemoCue and i-STAT, indicating that these devices yielded lower Hgb measurements than the lab-Hgb on average, whereas Rad-67 provided higher Hgb measurements than the lab-Hgb, on average, with a slightly higher mean difference within the population of lab-Hgb <100 g L^−1^ (Table 2). For all three POCT-Hgb devices, no LOA was within the allowable difference of within 4 g L^−1^. When considering secondary agreement thresholds, HemoCue demonstrated LOAs within 10 g L^−1^ from lab-Hgb for both the full population and those with lab-Hgb <100 g L^−1^, whereas i-STAT and Rad-67 did not have LOAs within any of the predetermined agreement thresholds. HemoCue had the narrowest LOA, and Rad-67 had the widest LOA of the three POCT-Hgb devices within both study populations. Subgroup analyses examining the effect of sex on agreement measures did not find any significant differences between each POCT-Hgb device and lab-Hgb (data not shown). Sensitivity analyses examining the influence of repeated measurements and protocol deviations did not change the conclusions of agreement obtained for each device, within both study populations (data not shown). Although differences in LOAs were noted when removing outliers (Supplementary Table S3), these did not affect conclusions about agreement and device interchangeability. Folded empirical distribution function curves presented findings consistent with LOA analyses (Supplementary Fig. S3).

**Table 2:** Limits of agreement between point-of-care and laboratory Hgb devices. CI, confidence interval; Lab-Hgb, laboratory-determined haemoglobin; POCT-Hgb, point-of-care testing of haemoglobin; RBC, red blood cell.

POCT-Hgb device	Mean difference (95% CI) (g L^−1^)	Lower limit (95% CI) (g L^−1^)	Upper limit (95% CI) (g L^−1^)
Full population			
HemoCue (*n*=1725)	–1.1 (–1.3, –0.9)	–9.6 (–10.0, –9.2)	7.5 (7.1, 7.8)
i-STAT (*n*=1711)	–3.1 (–3.4, –2.8)	–15.2 (–15.8, –14.7)	9.0 (8.4, 9.5)
Rad-67 (*n*=1312)	7.6 (6.8, 8.4)	–21.0 (–22.5, –19.5)	36.2 (34.7, 37.7)
Lab-Hgb <100 g L^−1^ (higher potential for RBC transfusions)			
HemoCue (*n*=680)	–0.8 (–1.1, –0.4)	–9.5 (–10.1, –8.9)	8.0 (7.3, 8.6)
i-STAT (*n*=671)	–2.4 (–2.9, –1.8)	–16.2 (–17.2, –15.2)	11.5 (10.5, 12.5)
Rad-67 (*n*=490)	12.9 (11.6, 14.1)	–14.7 (–17.0, –12.4)	40.5 (38.2, 42.8)

Disagreement proportions between each POCT-Hgb device and lab-Hgb, based on the three predetermined agreement thresholds, are presented in Table 3 for the full population and the population at highest risk of blood transfusion (lab-Hgb <100 g L^−1^). HemoCue had the lowest disagreement proportion of all three devices for both study populations. Disagreement proportions for Rad-67 were all >50%, using the 4 g L^−1^ and 7% thresholds, whereby >50% of Rad-67 measurements disagreed with the lab-Hgb results.

**Table 3:** Disagreement proportions between point-of-care and laboratory Hgb devices. Lab-Hgb, laboratory-determined haemoglobin; POCT-Hgb, point-of-care testing of haemoglobin.

POCT-Hgb device	Full population (*n*=1726)		Lab-Hgb <100 g L^−1^ (*n*=680)	
	*n*	% (95% CI)	*n*	% (95% CI)
Disagreement threshold: within 4 g L^−1^ when lab-Hgb <100 g L^−1^ and within 5% g L^−1^ when lab-Hgb ≥100 g L^−1^				
HemoCue	1725	11.9 (10.5, 13.6)	680	12.8 (10.4, 15.6)
i-STAT	1711	40.9 (38.6, 43.3)	671	51.3 (47.4, 55.1)
Rad-67	1312	72.7 (70.2, 75.1)	490	81.8 (78.1, 85.1)
Disagreement threshold: within 7% g L^−1^				
HemoCue	1725	5.0 (4.0, 6.2)	680	6.3 (4.7, 8.5)
i-STAT	1711	26.6 (24.5, 28.8)	671	35.2 (31.6, 38.9)
Rad-67	1312	63.3 (60.7, 65.9)	490	76.5 (72.5, 80.2)
Disagreement threshold: within 10 g L^−1^				
HemoCue	1725	2.0 (1.4, 2.8)	680	2.4 (1.4, 3.9)
i-STAT	1711	10.6 (9.2, 12.2)	671	11.0 (8.8, 13.7)
Rad-67	1312	51.9 (49.2, 54.6)	490	60.6 (56.1, 64.9)

**Fig 1:**
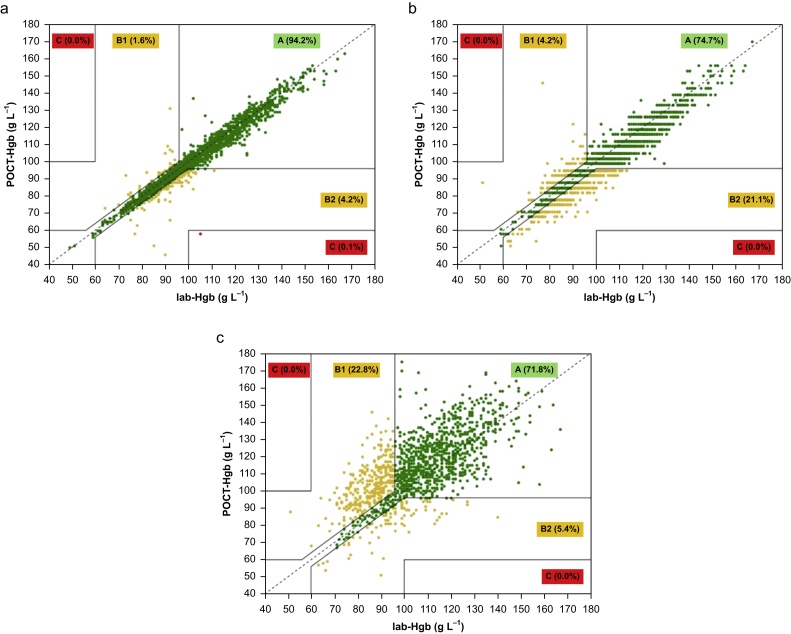
Error grid analyses. (a) HemoCue, (b) i-STAT, and (c) Rad-67. Zone A (green) represents low risk for potential transfusion errors, Zone B1 (yellow) represents moderate risk for under-transfusion, Zone B2 (yellow) represents moderate risk for over-transfusion, and Zone C (red) represents high risk for transfusion errors. The percentages indicated for each zone represent the proportion of measurements within that zone for the full population. Lab-Hgb, laboratory-determined haemoglobin; POCT-Hgb, point-of-care testing of haemoglobin.

Error grid analyses are reported in Figure 1. HemoCue had the highest proportion of observations within Zone A (clinically acceptable result), whereas Rad-67 had the lowest. HemoCue was associated with a 5.8% incidence of potentially clinically significant transfusion error (zone B), whereas i-STAT and Rad-67 were associated with 25.3% and 28.2%, respectively. There was a higher potential for over-transfusion with HemoCue (2.6–1) and i-STAT (5.0–1), whereas the potential for under-transfusion was higher with Rad-67 (1–4.1).

Cohen's kappa statistics are presented in Supplementary Table S4. HemoCue showed the best agreement beyond chance alone, with kappa statistics ranging from 0.77 to 0.88. i-STAT had kappa statistics slightly lower than those of HemoCue, ranging from 0.53 to 0.74. Rad-67 had the lowest kappa statistics, ranging from 0.33 to 0.47.

## Discussion

The PREMISE prospective cohort study examined 1735 blood samples collected in the operating room more than 17 months, encompassing a broad range of elective and urgent operations. Based on our main analyses, we observed that none of the three POCT-Hgb devices tested can be considered equivalent with lab-Hgb based on a 4 g L^−1^ threshold.^26^ HemoCue was the least likely to lead to transfusion errors with 5.8% of measurements, potentially leading to mistransfusion compared with 25.3% and 28.2% of Hgb measurements obtained by i-STAT and Rad-67, respectively. No device consistently over- or under-estimated lab-Hgb in the same direction. Although POCT-Hgb measurements with a lab-Hgb <100 g L^−1^ had slightly worse agreement with the lab-Hgb, conclusions about device accuracy did not change when compared with the full population.

**Fig 2:**
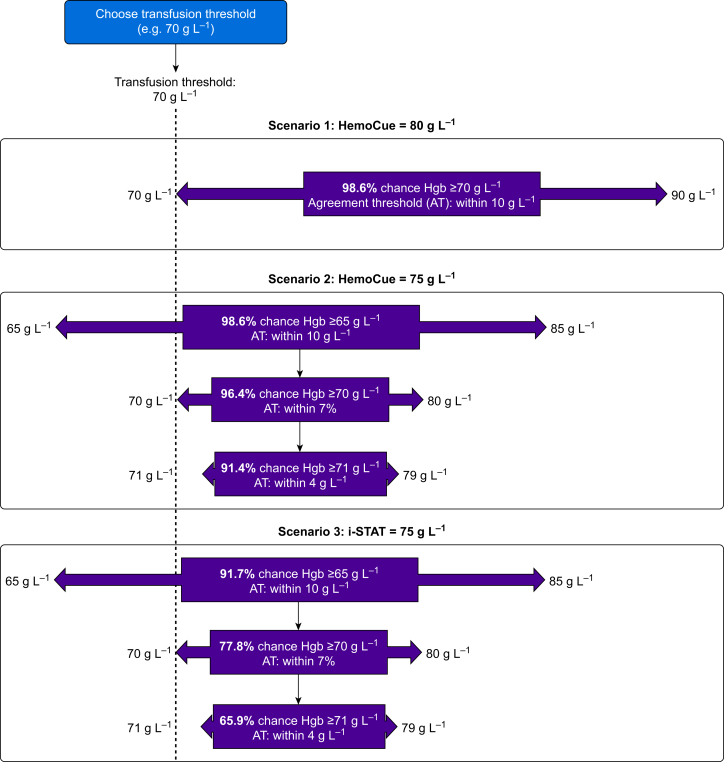
Clinical interpretation of study findings. AT, agreement threshold; Hgb, haemoglobin.

Of the three POCT-Hgb devices under investigation, HemoCue was the most accurate. More than 88% (95% CI 86.4–89.5%) of its measurements would be expected to be within 4 g L^−1^ from lab-Hgb, whereas 98% (95% CI 97.2–98.6%) would be within 10 g L^−1^. A clinician can use these probabilities to make transfusion decisions in the context of intraoperative blood-sparing strategies (Fig. 2). For example, with a transfusion threshold of 70 g L^−1^, a HemoCue reading of 80 g L^−1^ would imply that the true value lies between 70 and 90 g L^−1^ in 98.6% of cases, with only a 1.4% chance of error. As the measured Hgb approaches within 10 g L^−1^ of a chosen transfusion threshold, greater care is warranted because clinicians might need to validate results with the central laboratory in stable patients and should consider that use of HemoCue can lead to over-transfusion at a ratio of 2.6 to 1.

These findings are consistent with previous studies in which HemoCue was found to be generally more accurate than co-oximetry devices.^11^^,^^12^^,^^36^^,^^37^ HemoCue was found to be more accurate than i-STAT, which is an important confirmatory finding as HemoCue measures Hgb directly using a spectrophotometric method,^17^ whereas i-STAT estimates Hgb indirectly based on measured Hct.^18^ Our study is novel and important in testing a range of commonly used POCT-Hgb technologies. Previous studies had relatively small sample sizes and rarely accounted for repeated measurements, an important methodological consideration.^11^^,^^12^

Rad-67 was chosen as a co-oximetry device for examination of accuracy as it provides spot-check estimates of Hgb concentrations to be comparable with HemoCue and i-STAT. To our knowledge, this is the first study to examine its accuracy in the operating room.^38^^,^^39^ In the intraoperative setting, other groups have examined the accuracy of Radical-7 (Masimo, Irvine, CA, USA), a noninvasive pulse co-oximetry device that provides a continuous reading of Hgb, and Pronto-7 (Masimo, Irvine, CA, USA), an alternate co-oximetry device that provides intermittent readings of Hgb.^12^ Other groups have also evaluated trends in Hgb readings, which has been argued to be as important as accurate Hgb measurements.^40^^,^^41^

One of the primary strengths of this study is its large sample size, which provides a more precise LOA, evidenced by the narrow 95% CI observed.^21^ It fills a critical knowledge gap calling for large adequately powered studies^13^^,^^27^ pertaining to POCT-Hgb accuracy within the range of Hgb measurements in which RBC transfusion is most probable (<100 g L^−1^). Use of statistical methods to account for repeated measures when multiple POCT-Hgb measurements were performed within a single operation is an important element of this work.^20^^,^^21^ This was important, as the inclusion of multiple measurements within a single operation could result in an overestimation of agreement between POCT-Hgb and lab-Hgb given the correlation between repeated measurements. Moreover, multiple statistical methods were used to examine the outcomes, all of which were complementary and supported the identification of patterns in Hgb measurement differences. Finally, this study was designed to be pragmatic, inclusive, and observational to have no influence on concurrent clinical care. This strategy made it possible to enrol the most eligible participants, thus increasing generalisability.

This study has some limitations. Firstly, data were missing in 24% of Rad-67 measurements, which is unlikely to be random. Given that Hgb measurement was the main study outcome, use of statistical techniques for data imputation was not appropriate. Secondly, study hours were designed to capture the majority of POCT-Hgb use, but many measurements were performed outside of study hours. Although certain types of patients are more likely to have surgery after hours (e.g. penetrating trauma), this is unlikely to have meaningfully affected results, as the study captured a large proportion of emergency cases. Thirdly, only three types of POCT-Hgb devices were studied, chosen to represent the most commonly used devices in North America and because of their availability and affordability. Fourthly, the accuracy of table-top blood gas analysers was not examined, which are also occasionally used in operating rooms in lieu of portable POCT devices. Evidence suggests that these are no more accurate than other methods that estimate Hgb from Hct measurements, such as i-STAT, although this remains a limitation of this work.^11^ Fifthly, although HemoCue can be used with capillary blood samples, this study only included samples from arterial or central venous catheters. Therefore, results pertaining to HemoCue cannot be assumed to apply to capillary samples without further prospective evaluation. Sixthly, study results have not been adjusted for clinical factors that might interfere with device accuracy (e.g. use of vasopressors or blood transfusions). The effect of potentially interfering factors will form the basis of secondary analyses to be reported separately.^14^ Finally, although the lab-Hgb results are generally considered the gold standard by clinicians, these are not perfect measures of Hgb, as some measurement error is also acceptable under IQMH definitions.^26^

In conclusion, point-of-care Hgb measurements on arterial and central venous catheter blood samples can vary widely and lead to considerable differences when compared with laboratory Hgb results. Therefore, none of the devices studied can be considered interchangeable with laboratory measurements and should be used with caution when determining the need for intraoperative transfusions. Under specific intraoperative conditions leading to urgent transfusion decision-making, HemoCue is likely the most suitable point-of-care device given that it yielded Hgb measurements within 10 g L^−1^ compared with laboratory measurements in 98% of intraoperative blood samples.

## Authors’ contributions

Writing of the protocol: KB, LM, AW, DAF, GM

Writing of the statistical analysis plan (SAP): KB, RM, FMC, GM

Study-related activities: KB, LM

Overseeing study-related activities: AW, GM

Recruitment: KB, LM

Data collection: KB, LM

Statistical analysis: KB, RM,

Overseeing statistical analyses: TR, DAF, GM

Writing of the final manuscript: KB, FMC, DAF, GM

Study design: DIM, TR, JS, DAF, GM

Revising the first drafts of the protocol: DIM, CW, TR, AT, JS, JP, FMC, JH

Revising the first drafts of the SAP: DIM, TR

Revising the first drafts of the final manuscript: DIM, CW, TR, AT, JS, JP, JH

Providing expertise during the study design phase: CW, AT, JP, FMC

Principal Investigator: GM

All authors, external and internal, had full access to all data (including statistical reports and tables) in the study and agree to be accountable for the integrity of the data and the accuracy of the data analysis. The guarantor accepts full responsibility for the work and the conduct of the study, had access to the data, and controlled the decision to publish. The corresponding author attests that all listed authors meet authorship criteria and that no others meeting the criteria have been omitted.

## Declaration of interest

The authors declare that they have no conflict of interest. Specifically, no device manufacturer was involved in funding this study or in any portion of this study.
