# Supplementary material for: Evaluation of point-of-care haemoglobin measurement accuracy in surgery (PREMISE) and implications for transfusion practice: a prospective cohort study
Source: Br J Anaesth. 2025 Jan 9;134(2):341–9. doi: 10.1016/j.bja.2024.09.033 (PMC11775836; doi:10.1016/j.bja.2024.09.033)
Supplement: Multimedia component 1 [file mmc1.docx]

**Supplemental Online Content**

Brousseau et al. An evaluation of haemoglobin accuracy in surgery (PREMISE) and implications for transfusion practice: prospective cohort study

[**eTable 1.** STARD Checklist](#_Toc161832194)

[**eTable 2.** List of protocol deviations](#_Toc161832195)

[**eTable 3.** Limits of agreement estimates when outliers are removed from analysis](#_Toc161832196)

[**eTable 4.** Cohen’s κ statistic results](#_Toc161832197)

[**eFigure 1.** Flow of participants](#_Toc171077996)

[**eFigure 2.** Limits of agreement plots for the full population and for lab-Hgb <100 g L^-1^](#_Toc171077997)

[**eFigure 3.** Mountain plots for the full population and for lab-Hgb <100 g L^-1^](#_Toc171077998)

**eReferences**

**eTable 1.** STARD Checklist

|  | **Section & Topic** | **No** | **Item** | **Reported on page #** |
| --- | --- | --- | --- | --- |
|  | **TITLE OR ABSTRACT** |  |  |  |
|  |  | **1** | Identification as a study of diagnostic accuracy using at least one measure of accuracy (such as sensitivity, specificity, predictive values, or AUC) | NA (method comparison study) |
|  | **ABSTRACT** |  |  |  |
|  |  | **2** | Structured summary of study design, methods, results, and conclusions  (for specific guidance, see STARD for Abstracts) | 2 |
|  | **INTRODUCTION** |  |  |  |
|  |  | **3** | Scientific and clinical background, including the intended use and clinical role of the index test | 3 |
|  |  | **4** | Study objectives and hypotheses | 3 |
|  | **METHODS** |  |  |  |
|  | *Study design* | **5** | Whether data collection was planned before the index test and reference standard were performed (prospective study) or after (retrospective study) | 4 |
|  | *Participants* | **6** | Eligibility criteria | 4 |
|  |  | **7** | On what basis potentially eligible participants were identified  (such as symptoms, results from previous tests, inclusion in registry) | 4 |
|  |  | **8** | Where and when potentially eligible participants were identified (setting, location and dates) | 4 |
|  |  | **9** | Whether participants formed a consecutive, random or convenience series | 4 |
|  | *Test methods* | **10a** | Index test, in sufficient detail to allow replication | 4, 5 |
|  |  | **10b** | Reference standard, in sufficient detail to allow replication | 4, 5 |
|  |  | **11** | Rationale for choosing the reference standard (if alternatives exist) | 5 |
|  |  | **12a** | Definition of and rationale for test positivity cut-offs or result categories  of the index test, distinguishing pre-specified from exploratory | 6 |
|  |  | **12b** | Definition of and rationale for test positivity cut-offs or result categories  of the reference standard, distinguishing pre-specified from exploratory | 6 |
|  |  | **13a** | Whether clinical information and reference standard results were available  to the performers/readers of the index test | 4,5 |
|  |  | **13b** | Whether clinical information and index test results were available  to the assessors of the reference standard | 4,5 |
|  | *Analysis* | **14** | Methods for estimating or comparing measures of diagnostic accuracy | 5-7 |
|  |  | **15** | How indeterminate index test or reference standard results were handled | 7 |
|  |  | **16** | How missing data on the index test and reference standard were handled | 7 |
|  |  | **17** | Any analyses of variability in diagnostic accuracy, distinguishing pre-specified from exploratory | 6 |
|  |  | **18** | Intended sample size and how it was determined | 7 |
|  | **RESULTS** |  |  |  |
|  | *Participants* | **19** | Flow of participants, using a diagram | eFigure 1 |
|  |  | **20** | Baseline demographic and clinical characteristics of participants | 8, Table 1 |
|  |  | **21a** | Distribution of severity of disease in those with the target condition | NA |
|  |  | **21b** | Distribution of alternative diagnoses in those without the target condition | NA |
|  |  | **22** | Time interval and any clinical interventions between index test and reference standard | 4, 5 |

| **Section & Topic** | **No** | **Item** | **Reported on page #** |
| --- | --- | --- | --- |
| *Test results* | **23** | Cross tabulation of the index test results (or their distribution)  by the results of the reference standard | Table 3 |
|  | **24** | Estimates of diagnostic accuracy and their precision (such as 95% confidence intervals) | 8, 9, Table 2, Figure 1 |
|  | **25** | Any adverse events from performing the index test or the reference standard | 8 |
| **DISCUSSION** |  |  |  |
|  | **26** | Study limitations, including sources of potential bias, statistical uncertainty, and generalisability | 11, 12 |
|  | **27** | Implications for practice, including the intended use and clinical role of the index test | 10, Figure 2 |
| **OTHER INFORMATION** |  |  |  |
|  | **28** | Registration number and name of registry | 13 |
|  | **29** | Where the full study protocol can be accessed | 4 |
|  | **30** | Sources of funding and other support; role of funders | 13 |

Abbreviation: NA, Not applicable; STARD, Standard for Reporting Diagnostic accuracy studies.

**eTable 2.** List of protocol deviations

| **Deviation** | **N (%)**  **(n=1726)** |
| --- | --- |
| No general or neuraxial anaesthesia | 5 (0.3) |
| Age <18 years | 3 (0.2) |
| Anaesthesiologist not available to answer questionnaire | 65 (3.8) |
| Rad-67 done more than five minutes after blood draw | 17 (1.0) |
| Different blood sample used for POCT-Hgb testing | 14 (0.8) |

Abbreviations: POCT-Hgb, Point-of-care testing of haemoglobin.

**eTable 3.** Limits of agreement estimates when outliers are removed from analysis

|  | n outliers removed | Mean bias  (g L^-1^) | Lower limit  (g L^-1^) | 95% CI of the lower limit | Upper limit  (g L^-1^) | 95% CI of the upper limit |
| --- | --- | --- | --- | --- | --- | --- |
| **Full population** | | | | | | |
| HemoCue  (n=1721) | 4 | -1.0 | **-8.5** | -8.8, -8.1 | **6.4** | 6.0, 6.7 |
| i-STAT  (n=1708) | 3 | -3.2 | **-14.6** | -15.1, -14.0 | **8.1** | 7.6, 8.6 |
| Rad-67  (n=1308) | 4 | 7.6 | **-20.2** | -21.7, -18.7 | **35.4** | 33.9, 36.8 |
| **Lab-Hgb <100 g L^-1^ (higher potential for RBC transfusions)** | | | | | | |
| HemoCue  (n=675) | 5 | -0.7 | **-7.5** | -8.0, -7.0 | **6.2** | 5.7, 6.7 |
| i-STAT  (n=668) | 3 | -2.6 | **-14.7** | -15.6, -13.8 | **9.6** | 8.7, 10.5 |
| Rad-67  (n=486) | 4 | 12.8 | **-13.1** | -15.3, -10.9 | **38.8** | 36.6, 40.9 |

Abbreviations: CI, Confidence interval; Lab-Hgb, Laboratory-determined haemoglobin; RBC, Red blood cells.

**eTable 4.** Cohen’s κ statistic results

| **Agreement threshold** | **Cohen’s κ^a^ (95% CI)** | | |
| --- | --- | --- | --- |
|  | **i-STAT**  **(n=1711)** | **HemoCue**  **(n=1725)** | **Rad-67**  **(n=1312)** |
| <100 g L^-1^ | 0.74 (0.71, 0.78) | 0.88 (0.86, 0.90) | 0.47 (0.42, 0.52) |
| <90 g L^-1^ | 0.72 (0.68, 0.76) | 0.85 (0.82, 0.88) | 0.34 (0.28, 0.40) |
| <85 g L^-1^ | 0.72 (0.67, 0.77) | 0.84 (0.80, 0.87) | 0.34 (0.26, 0.42) |
| <80 g L^-1^ | 0.65 (0.59, 0.71) | 0.83 (0.78, 0.88) | 0.34 (0.23, 0.45) |
| <75 g L^-1^ | 0.63 (0.54, 0.72) | 0.77 (0.68, 0.86) | 0.33 (0.18, 0.48) |
| <70 g L^-1^ | 0.53 (0.41, 0.66) | 0.84 (0.73, 0.94) | 0.38 (0.14, 0.62) |

Abbreviation: CI, Confidence interval.

^a^ κ statistic <0.00 represents poor agreement, 0.00 to 2.00 is slight agreement, 0.21 to 0.40 is fair agreement, 0.41 to 0.60 is moderate agreement, 0.61 to 0.80 is substantial agreement, and 0.81 to 1.00 is almost perfect agreement.^1,2^

**eFigure 1.** Flow of participants

**
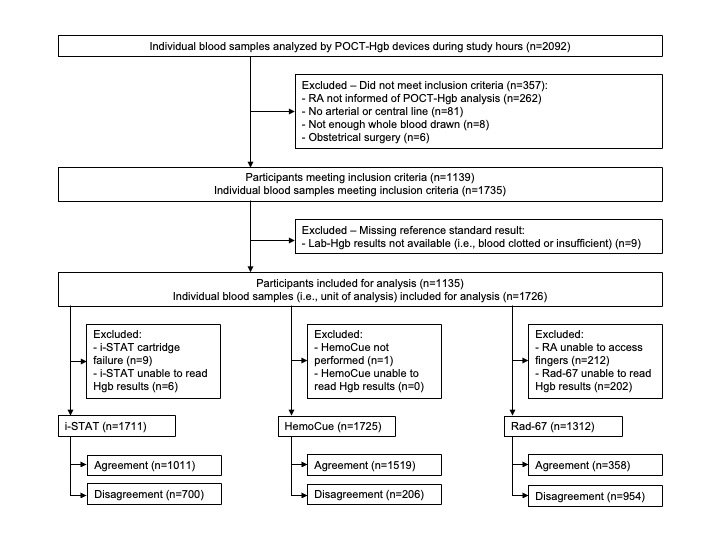
**

Abbreviations: Hgb, Haemoglobin; Lab-Hgb, Laboratory-determined haemoglobin; POCT-Hgb, Point-of-care testing of haemoglobin; RA, Research assistant.

Agreement and disagreement incidence for each POCT-Hgb device are based on the Institute for Quality Management in Healthcare (IQMH) definition.

**eFigure 2.** Limits of agreement plots for the full population and for lab-Hgb <100 g L^-1^

| **A HemoCue within the full population** | **B Hemocue when Lab-Hgb <100 g L^-1^** |
| --- | --- |
| 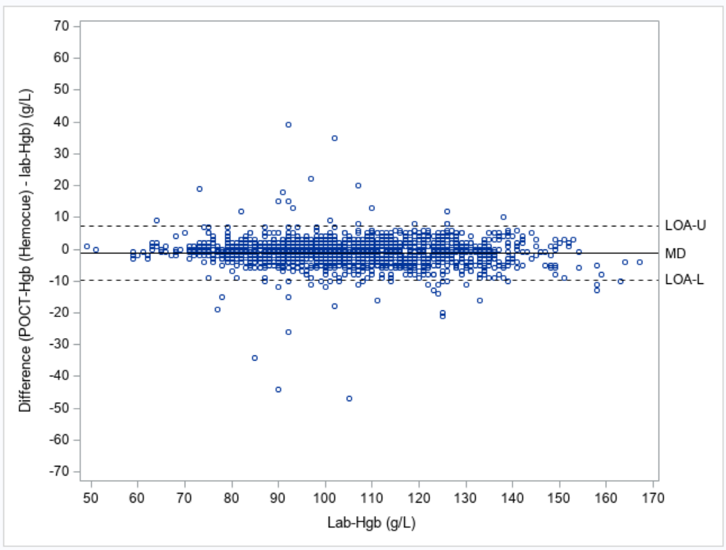 | 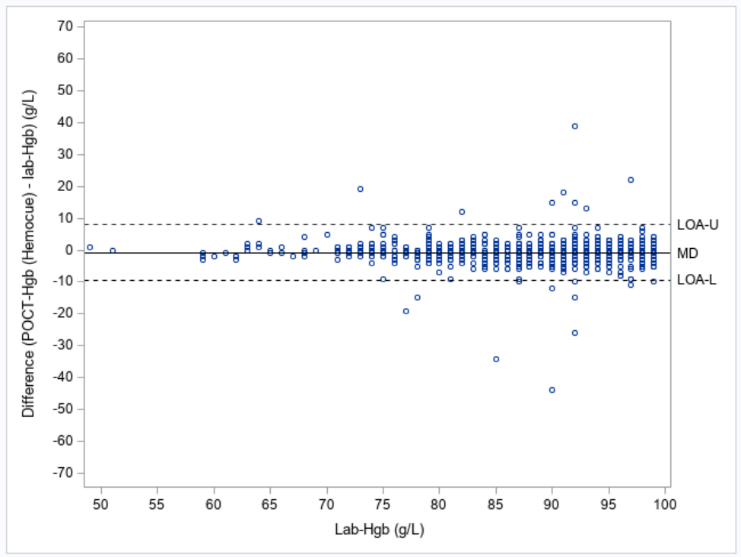 |
| **C i-STAT within the full population** | **D i-STAT when Lab-Hgb <100 g L^-1^** |
| 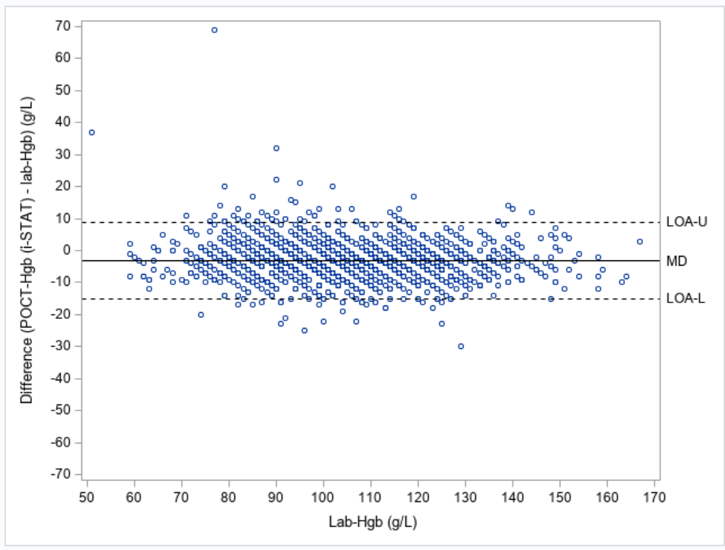 | 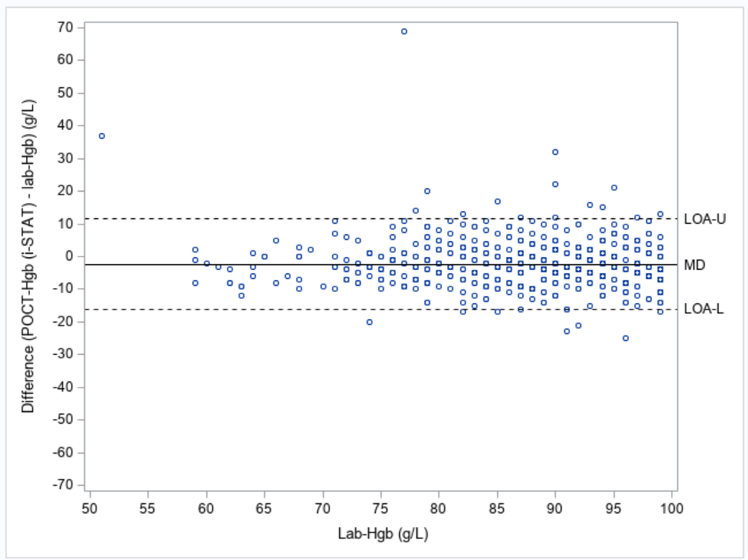 |
| **E Rad-67 within the full population** | **F Rad-67 when Lab-Hgb <100 g L^-1^** |
| 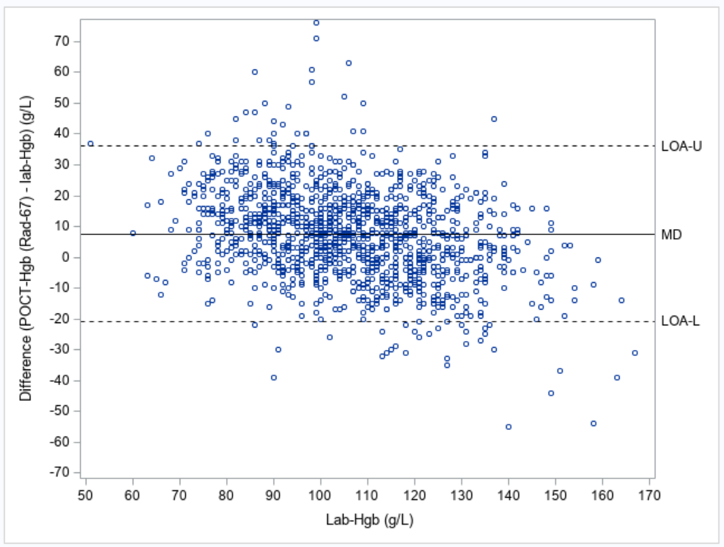 | 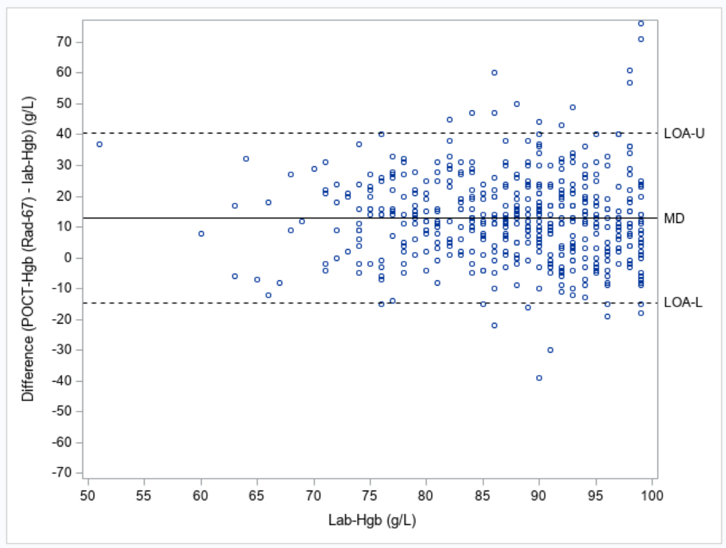 |

Abbreviations: Lab-Hgb, Laboratory-determined haemoglobin; LOA-L, Lower limit of agreement; LOA-U, Upper limit of agreement; MD, Mean difference; POCT-Hgb, Point-of-care testing of haemoglobin.

**eFigure 3.** Mountain plots for the full population and for lab-Hgb <100 g L^-1^

| **A Full population** |
| --- |
| 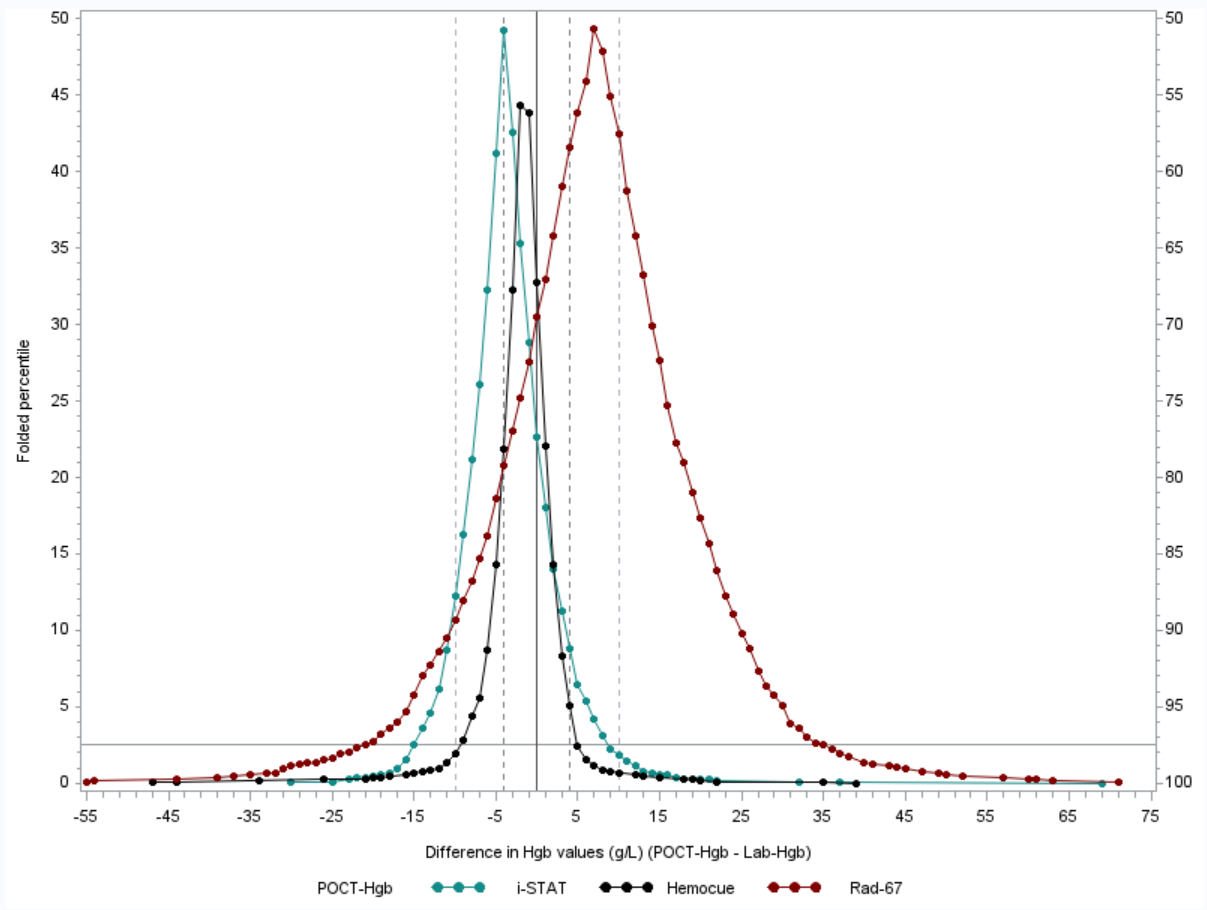 |
| **B Lab-Hgb <100 g L^-1^** |
| 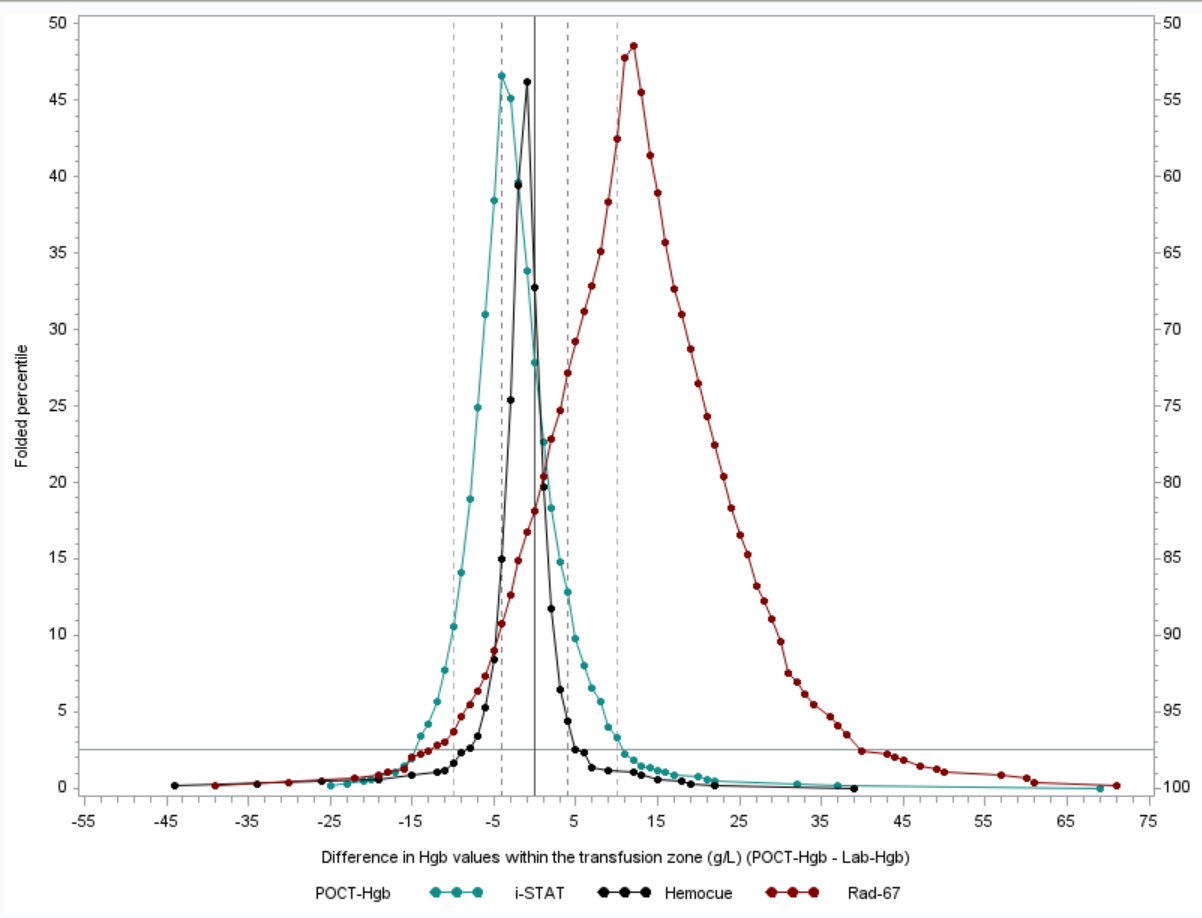 |

Abbreviations: Lab-Hgb, Laboratory-determined haemoglobin; POCT-Hgb, Point-of-care testing of haemoglobin.

**eReferences**

1. Rigby AS. Statistical methods in epidemiology. v. Towards an understanding of the kappa coefficient. *Disabil Rehabil*. 2000;22(8):339-344. doi:10.1080/096382800296575

2. Landis JR, Koch GG. The Measurement of Observer Agreement for Categorical Data. *Biometrics*. 1977;33:159-174. doi:10.2307/2529310
